# Preventive effect of low-dose landiolol on postoperative atrial fibrillation study (PELTA study)

**DOI:** 10.1007/s11748-020-01364-9

**Published:** 2020-05-05

**Authors:** Konosuke Sasaki, Kiichiro Kumagai, Kay Maeda, Masatoshi Akiyama, Koki Ito, Satoshi Matsuo, Shintaro Katahira, Tomoyuki Suzuki, Yusuke Suzuki, Yu Kaiho, Yumi Sugawara, Ichiro Tsuji, Yoshikatsu Saiki

**Affiliations:** 1 Division of Cardiovascular Surgery, Tohoku University Graduate School of Medicine, 1-1 Seiryo-machi, Aoba-ku, Sendai, 980-8574 Japan; 2 Research Division of Sciences for Aortic Disease, Tohoku University Graduate School of Medicine, Sendai, Japan; 3 Division of Epidemiology, Department of Health Informatics and Public Health, Tohoku University School of Public Health, Graduate School of Medicine, Sendai, Japan

**Keywords:** Landiolol hydrochloride, Postoperative atrial fibrillation, Prophylactic administration, Low dose, Cardiovascular surgery

## Abstract

**Objective:**

To investigate the efficacy of prophylactic administration of low-dose landiolol on postoperative atrial fibrillation (POAF) in patients after cardiovascular surgery.

**Methods:**

Consecutive 150 patients over 70 years of age who underwent cardiovascular surgery for valvular, ischemic heart, and aortic diseases were enrolled in this single-center prospective randomized control study from 2010 to 2014. They were assigned to three treatment groups: 1γ group (landiolol at 1 μg/kg/min), 2γ group (landiolol at 2 μg/kg/min), or control group (no landiolol). In the two landiolol groups, landiolol hydrochloride was intravenously administered for a period of 4 days postoperatively. Electrocardiography was continuously monitored during the study period, and cardiologists eventually assessed whether POAF occurred or not.

**Results:**

POAF occurred in 24.4% of patients in the control group, 18.2% in 1γ group, and 11.1% in 2γ group (*p* = 0.256). Multivariate logistic regression analysis showed that the incidence of POAF tended to decrease depending on the dose of landiolol (trend-*p* = 0.120; 1γ group: OR = 0.786, 95% CI 0.257–2.404; 2γ group: OR = 0.379, 95% CI 0.112–1.287). Subgroup analysis showed a significant dose-dependent reduction in POAF among categories of female sex, non-use of angiotensin II receptor blockers (ARBs) before surgery, and valve surgery (each trend-*p* = 0.02, 0.03, and 0.004).

**Conclusions:**

These findings indicate that prophylactic administration of low-dose landiolol may not be effective for preventing the occurrence of POAF in overall patients after cardiovascular surgery, but the administration could be beneficial to female patients, patients not using ARBs preoperatively, and those after valvular surgery.

## Introduction

Postoperative atrial fibrillation (POAF) is one of the most common complications after cardiovascular surgery. The incidence of POAF varies widely from approximately 18–64% [1, 2] and is greater among elderly patients [3]. A higher prevalence of POAF has been associated with advanced age, obesity, preoperative complications such as respiratory failure and renal dysfunction, and cardiac dysfunction [3, 4]. Left ventricular diastolic dysfunction causes atrial and ventricular pressure loads, which leads to atrial remodeling and is closely involved in its occurrence. POAF is globally known to increase morbidity after cardiovascular surgery, and a meta-analysis report on post-coronary artery bypass grafting (CABG) has shown that POAF increased in-hospital and long-term mortality [5]. Another meta-analysis found that preoperative use of β-blockers reduced the occurrence of new onset atrial fibrillation [6]. Guidelines published by the American College of Cardiology/American Heart Association/European Society of Cardiology and the American Association for Thoracic Surgery recommend against withdrawal of preoperative oral β-blockers medication and for their continuous usage during the perioperative period to reduce the incidence of POAF in patients undergoing cardiac surgery (recommendation class I, level of evidence A) [7, 8].

Landiolol hydrochloride, an ultra-short acting β1-selective blocker, has been known to exert a clinically relevant negative chronotropic action without any negative inotropic effects at low dose. Landiolol was effective in treating the patients developing POAF after open-heart surgery. A retrospective study documented the preventive effect of titrated low-dose landiolol administration on atrial fibrillation after CABG [9]. Several randomized controlled trials (RCTs) showed that landiolol has a prophylactic effect against POAF onset. A meta-analysis of the 6 RCTs evaluated the effectiveness of landiolol administration for the prevention of POAF after cardiac surgery [10], finding that 5 RCTs highlighted the effect of landiolol on post-CABG POAF, while the other focused exclusively on valvular heart surgery. Moreover, in most of these RCTs, the dose of landiolol was more than 2 μg/kg/min and was titrated up as needed to maintain a low heart rate. Therefore, there is limited data available to evaluate the effect of adequate standard dose of landiolol on the outcomes after cardiovascular surgery in general, including heart valve and aortic surgery. In addition, the effectiveness of low-dose of landiolol for POAF prevention after cardiovascular surgery remains inconclusive.

This study, termed the preventive effect of low-dose landiolol on postoperative atrial fibrillation (PELTA) study, was designed to investigate the efficacy of low-dose landiolol hydrochloride for prevention of POAF in patients undergoing cardiovascular surgery, including CABG, valvular, and aortic surgery.

## Patients and methods

This was a single-center, prospective randomized controlled, open-label, and parallel study conducted from April 2010 to June 2014. All patients provided written informed consent before initiating the study enrollment. This study was approved by the Institutional Review Board at Tohoku University Graduate School of Medicine (first approval number 2009-483, latest approval 2012-1-97) and was conducted in accordance with the ethical principles of the Declaration of Helsinki. The study protocol was registered at the University Hospital Medical Information Network (UMIN 000003378).

After screening for eligibility criteria for this study, consecutive patients aged higher or equal to 70 years who underwent elective cardiovascular surgery for valvular, ischemic heart, and aortic diseases were enrolled. The cut-off age of 70 years old was determined based on our own analysis of risk factors for POAF when we participated in the JL-KNIGHT study (data not shown) [11]. Exclusion criteria were as follows: patients with preoperative atrial fibrillation; hemoglobin A1c ≥ 8.0%; severe asthma, defined as forced expiratory volume in 1 s (FEV1.0) under 1000 ml; allergy to landiolol hydrochloride; emergency surgery; or judged ineligible for this study by physicians.

### Study protocol

**Fig. 1:**
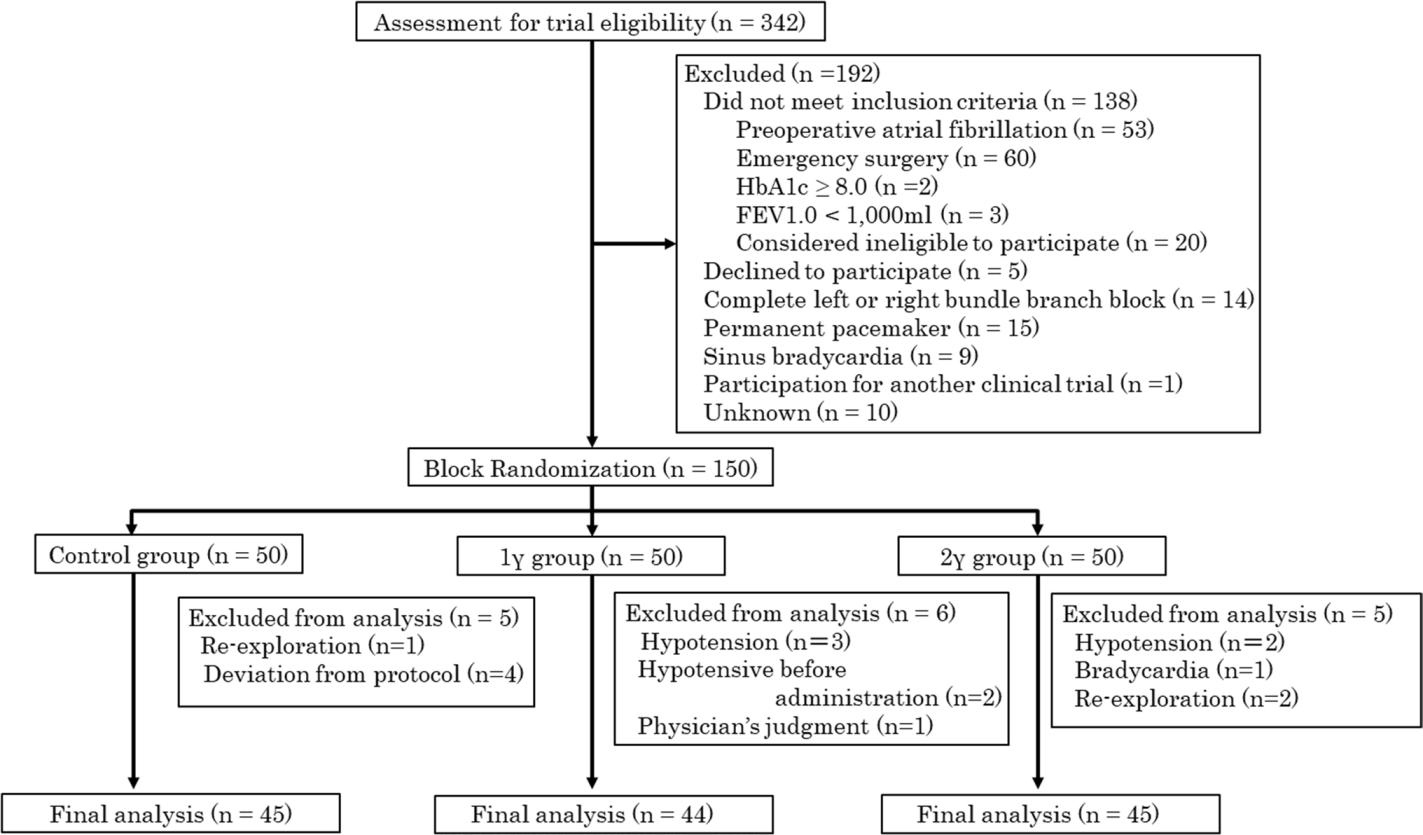
Clinical trial flowchart. PELTA study was composed of 150 patients who underwent cardiovascular surgery. *HbA1c* hemoglobin A1c, *FEV1.0* forced expiratory volume in 1 s, *γ* μg/kg/min

The eligible patients were assigned to one of the following 3 treatment groups (50 patients in each group): 1γ group (landiolol at 1 μg/kg/min), 2γ group (landiolol at 2 μg/kg/min), or control group (no landiolol). These two low doses were selected according to the findings from a pilot study. To balance the proportion of surgical procedures in each group, the allocation was performed through block randomization with the block sizes of three by one researcher who was blinded to patients’ clinical information. Patients were blinded to the assigned treatment in this single-blind study. The consolidated standards of reporting trials flow diagram shows the randomization and patient flow throughout the trial (Fig. 1). All patients were admitted to the intensive care unit (ICU) after the operation. In the 1γ and 2γ groups, landiolol hydrochloride was intravenously administered soon after ICU admission and continued for 4 days. ICU staff continuously monitored the patients’ electrocardiogram. POAF was defined as continuous atrial fibrillation sustained for more than 5 min and was eventually diagnosed by cardiologists. When a patient who had been assigned to landiolol group developed bradycardia, the temporary epicardial pacemaker was used to increase his or her heart rate to a reasonable level. Those patients were not excluded from this study unless they showed some other adverse events. The administration of oral β-blockers was prohibited during the study period. Echocardiography was performed on the preoperative day (Pre) and postoperative day (POD) 3. Laboratory blood sample data were obtained from Pre through to POD 4, since POAF’s peak incidence is from PODs 2–4. The primary endpoint was the occurrence of POAF between POD 1 and POD 4 in each group. The secondary endpoints were changes in echocardiographic data including left atrial diameter (LAD), left ventricular end-diastolic diameter (LVDd), and left ventricular ejection fraction (LVEF) to confirm the safety of low-dose landiolol administration in the acute phase after cardiovascular surgery; and blood biochemical data, including brain natriuretic peptide (BNP) as predisposing factor for POAF, and white blood cell count as an inflammatory marker.

### Statistical analysis

The sample size was estimated for the chi-squared test based on the assumption that the incidence of POAF would be 35% in the control group and 10% in the intravenous landiolol groups. It was calculated that 43 patients would be required for each study group with an α error of 5% and a power of 80%. Considering a drop rate of approximately 10%, 50 patients per group would be needed. This was an intention-to-treat analysis of all the patients enrolled. Continuous variables were expressed as mean ± standard deviation or median (interquartile range), where appropriate, after being tested for normality of distribution by the Kolmogorov–Smirnov test. These variables were compared between groups by means of one-way analysis of variance (ANOVA), followed by a post-hoc Tukey–Kramer method for pairwise comparisons of parametric parameters, or the Kruskal–Wallis test for nonparametric data. Repeated echocardiographic and blood data were compared by one-factor repeated measures ANOVA. Categorical variables were represented as absolute number or percentage, and Fisher’s exact test or the chi-squared test were used for comparison between groups. As for the occurrence of POAF, subgroup analyses were performed stratified by age, gender, comorbidities, preoperative medications, and types of cardiovascular surgery. The preventive effect of landiolol on the occurrence of POAF was assessed by multivariate logistic regression after adjustment for confounding candidates such as age, LVDd, and LVEF. The odds ratio (OR) and 95% confidence intervals (CI) were subsequently estimated for the two landiolol groups. The dose–response relationship of landiolol in POAF prevention was examined with the Cochran-Armitage test for trend. If a significant difference was found in the subgroup analysis, the OR of the subcategory and its 95% CI were calculated. Due to quasi-complete separation in the logistics regression, the prophylactic effect of landiolol in valvular surgeries was considering the 1γ and 2γ groups as the one composite landiolol group (1γ + 2γ). For all analyses, *p* < 0.05 was considered statistically significant. All statistical analyses were performed using the SAS statistical software package, version 9.4 (SAS Institute Inc, Cary, NC, USA).

## Results

One hundred ninety-two of 342 patients were excluded as shown in Fig. 1. After 150 patients were allocated randomly into the control, 1γ, and 2γ groups, 16 patients were withdrawn from this study due to the following reasons: side effects such as hypotension (3 patients in the 1γ group and 2 in the 2γ group) and bradycardia (1 TEVAR patient in the 2γ group without having a temporary epicardial pacemaker wire), immediate re-operation (1 patient in the control group and 2 in the 2γ group), difficulty in administering landiolol due to hypotension (2 patient in the 1γ group), deviation from the protocol (4 patients in the control group receiving variable doses of landiolol for tachycardia), and a physician’s judgement (1 patient in the 1γ group). Therefore, 134 patients were included in the final analysis.

### Patients’ characteristics and perioperative data

Baseline characteristics of patients in the 3 groups are listed in Table 1. All the preoperative characteristics were similar except for LVDd and LVEF. Post-hoc Tukey–Kramer testing showed a significant difference in LVDd between the control group and the two landiolol groups (*p* = 0.0036 between the control and 1γ groups, *p* = 0.008 between the control and 2γ groups). A significant difference in LVEF was observed between the control and 1γ groups (*p* = 0.016). However, these values were within the clinically normal range in the 3 groups. By using block randomization, the numbers of patients who had undergone valvular surgery and thoracic endovascular aortic repair (TEVAR) were almost equivalent, and the differences in the types of surgical procedure in each group were not statistically significant as shown in Table 2 (valvular surgery, *p* = 0.91; coronary artery bypass grafting, *p* = 0.32; aortic surgery, *p* = 0.37; TEVAR, *p* = 0.89). Among TEAVR, there were three patients who underwent TEVAR with de-branching via sternotomy. There were no significant differences among the three groups regarding, operating time, ICU stay, and lengths of the postoperative period.

**Table 1:** Preoperative baseline patient characteristics

	Control	1γ	2γ	*p* value
	(*n* = 45)	(*n* = 44)	(*n* = 45)	
Female	16 (35.6)	19 (43.2)	18 (40.0)	0.76
Age (years)	74.5 ± 3.9	76.6 ± 3.5	75.9 ± 5.0	0.06
Height (cm)	160.0 ± 9.1	156.2 ± 8.1	158.7 ± 8.8	0.12
Weight (kg)	59.6 ± 12.1	56.4 ± 10.5	56.6 ± 9.3	0.29
BMI (kg/m^2^)	23.1 ± 3.5	23.1 ± 3.6	22.4 ± 3.0	0.57
Comorbidities				
Hypertension	40 (88.9)	36 (81.8)	36 (80.0)	0.49
Diabetes mellitus	9 (20.0)	11 (25.0)	6 (13.3)	0.38
Dyslipidemia	18 (40.0)	15 (34.1)	23 (51.1)	0.25
CVD	12 (26.7)	12 (27.3)	12 (26.7)	1.00
Hemodialysis	3 (6.7)	4 (9.1)	1 (2.2)	0.38
Medications				
β-blockers	18 (40.0)	24 (54.5)	21 (46.7)	0.39
ARBs	30 (66.7)	29 (65.9)	26 (57.8)	0.63
CCB	27 (60.0)	30 (68.2)	23 (51.1)	0.26
Preoperative status				
HR (bpm)	76.7 ± 16.6	83.5 ± 22.2	75.5 ± 20.2	0.14
SBP (mmHg)	130.0 ± 15.3	130.9 ± 19.3	125.8 ± 18.9	0.38
DBP (mmHg)	76.4 ± 11.6	78.5 ± 11.7	75.4 ± 12.1	0.48
LAD (mm)	41.9 ± 7.0	40.3 ± 6.6	38.9 ± 7.4	0.14
LVDd (mm)	53.0 ± 9.0	49.0 ± 7.0	48.1 ± 6.4	0.01
LVEF (%)	60.6 ± 12.2	66.8 ± 7.7	64.7 ± 10.4	0.02
*E*/*e*′	15.1 ± 8.7	15.5 ± 5.3	15.9 ± 5.7	0.89
Blood biochemical data				
BNP (pg/ml)	56.6 (23.8–111.4)	56.9 (25.6–204.3)	47.7 (23.4–111.8)	0.74
Hb (g/dl)	12.2 ± 1.9	11.9 ± 1.6	11.7 ± 2.0	0.50
Ht (%)	36.2 ± 5.6	35.3 ± 4.5	35.1 ± 5.6	0.54
WBC (/μl)	5856 ± 2284	5902 ± 1360	5713 ± 1912	0.89
Plt (*10^4^/µl)	17.7 ± 5.8	16.8 ± 4.5	17.6 ± 5.7	0.72
AST (U/L)	24.0 ± 18.1	21.3 ± 16.5	25.4 ± 16.0	0.51
ALT (U/L)	15.3 ± 7.0	16.6 ± 14.9	19.9 ± 15.7	0.23
BUN (mg/dl)	23.2 ± 18.5	20.8 ± 9.3	19.3 ± 11.1	0.40
Cr (mg/dl)	1.0 (0.8–1.2)	1.1 (0.8–1.5)	0.9 (0.7–1.2)	0.29
TP (g/dl)	6.9 ± 0.6	6.8 ± 0.8	6.9 ± 0.7	0.76
LDH (IU/L)	203.2 ± 61.2	202.8 ± 72.7	211.2 ± 55.1	0.78
CK (IU/L)	69 (47–93)	54 (39–78)	62 (38–88)	0.31
CK-MB (IU/L)	10 (7–12)	9 (6–11)	9 (6–12)	0.6

Data are given as *n* (%), mean ± standard deviation or median (interquartile range). Fisher’s exact test was used to compare the number of patients undergoing hemodialysis

*γ* μg/kg/min, *BMI* body mass index, *CVD* cerebrovascular disease, *ARBs* angiotensin II receptor blockers, *CCB* calcium channel blocker, *HR* heart rate, *SBP* systolic blood pressure, *DBP* diastolic blood pressure, *LAD* left atrial diameter, *LVDd* left ventricular end-diastolic diameter, *LVEF* left ventricular ejection fraction, *BNP* brain natriuretic peptide, *Hb* hemoglobin, *Ht* hematocrit, *WBC* white blood cell, *Plt* platelet, *AST* aspartate aminotransferase, *ALT* alanine aminotransferase, *BUN* blood urea nitrogen, *Cr* creatinine, *TP* total protein, *LDH* lactate dehydrogenase, *CK* creatine phosphorus kinase, *CK-MB* creatine kinase MB

**Table 2:** Perioperative data

	Control	1γ	2γ	*p* value
	(*n* = 45)	(*n* = 44)	(*n* = 45)	
Operative data				
Surgical procedure				
Valve	16 (35.6)	17 (38.6)	18 (40.0)	0.91
CABG	9 (14.9)	6 (13.6)	4 (8.9)	0.32
Aorta	5 (11.1)	8 (18.2)	10 (22.2)	0.37
TEVAR	15 (33.3)	13 (29.5)	13 (28.9)	0.89
Operating time (min)	454.4 ± 230.9	500.6 ± 290.2	454.0 ± 232.7	0.61
Postoperative data				
ICU stay (days)	6.0 (4.0–10)	7.0 (4.0–13.0)	6.0 (4.0–8.0)	0.75
Lengths of postoperative period (days)	26.0 (19.0–47.0)	32.0 (21.0–56.0)	29.0 (22.0–45.0)	0.48

Data are given as *n* (%), mean ± standard deviation or median (interquartile range)

*γ* μg/kg/min, *CABG* coronary artery bypass grafting, *TEVAR* thoracic endovascular aortic repair, *ICU* intensive care unit

### Preventive effect of landiolol on POAF

**Fig. 2:**
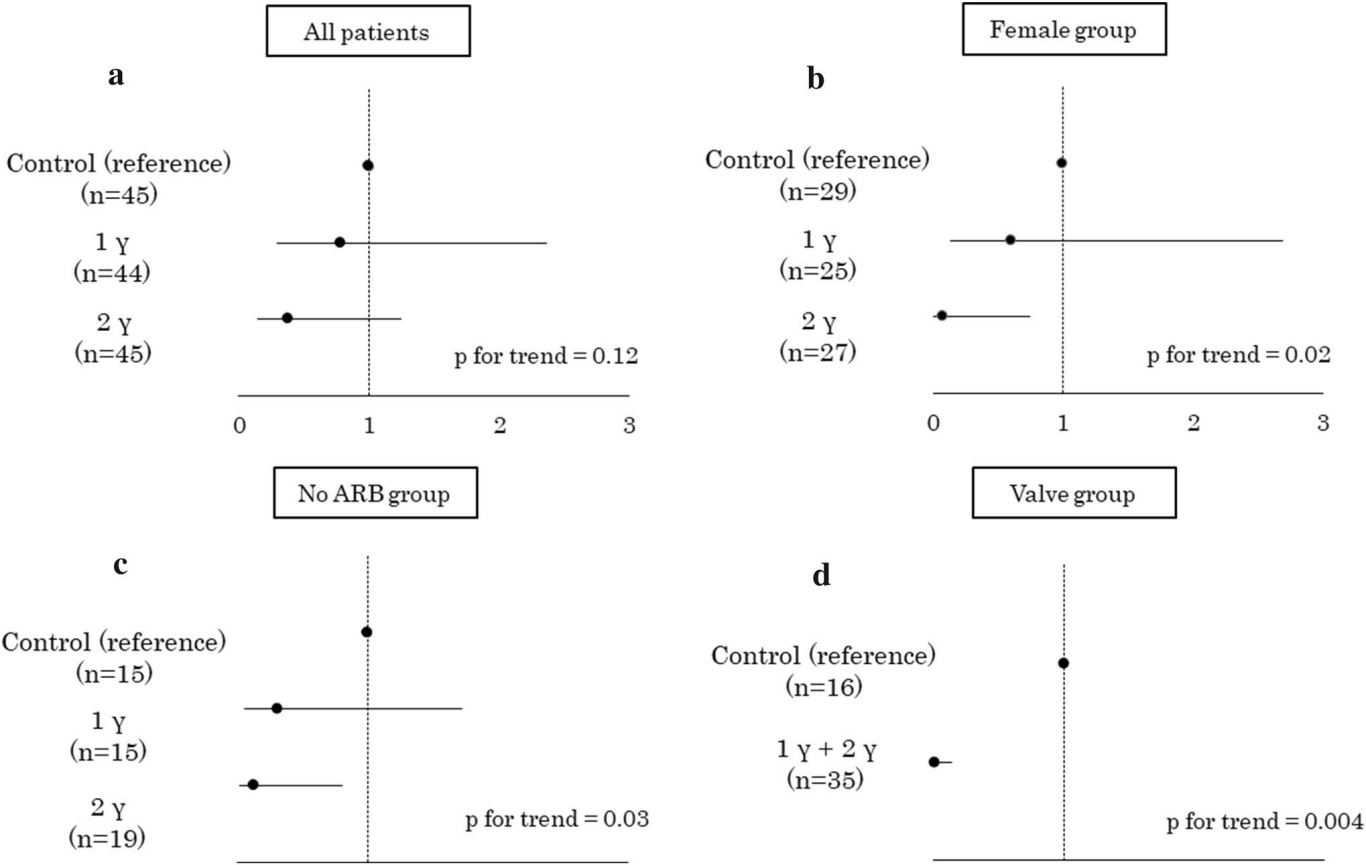
Preventive effects of landiolol for postoperative atrial fibrillation among all patients and subgroups. The black dots and bars represent the odds ratio and 95% confidence intervals, respectively. **a** All patients, **b** female patients, **c** patients not using ARBs prior to cardiovascular surgeries, **d** patients who underwent valvular surgery. Multivariate logistics regression was conducted after adjustment for age, left ventricular end-diastolic diameter, and left ventricular ejection fraction. The dose–response relationship was examined with the Cochran–Armitage test for trend. Due to quasi-complete separation in the logistics regression, the prophylactic effect of landiolol on postoperative atrial fibrillation after valvular surgeries was investigated in a composite 1γ + 2γ group. *γ* μg/kg/min, *ARBs* angiotensin II receptor blockers

Table 3 presents the incidence of POAF in each assigned group and patient category. POAF occurred in 24.4%, 18.2% and 11.1% of patients in the control, 1γ, and 2γ groups, respectively, with no significant difference among them (*p* = 0.26). There were no significant differences in POAF incidence among the three age categories; however, of subcategories, female sex, absence of preoperative angiotensin II receptor blockers (ARBs) use, and undergoing valvular surgery were significantly associated with a reduction in the incidence of POAF after landiolol administration (*p* = 0.03, 0.02, and < 0.01, respectively). Figure 2a shows the ORs and 95% CIs for the control and two landiolol groups estimated by multivariate logistics regression. The incidence of POAF tended to decrease in a landiolol dose-dependent manner [OR 0.79 (0.26–2.40) in the 1γ group and 0.38 (0.11–1.29) in the 2γ groups, *p* for trend = 0.12]. A significant preventive effect of landiolol against POAF was observed in female patients [OR 0.08 (0.01–0.75) in the 2γ group, Fig. 2b], patients not using ARBs preoperatively [OR 0.12 (0.02–0.81) in the 2γ group, Fig. 2c], and patients undergoing valvular surgery group [OR 0.002 (< 0.001–0.134) in the 1γ + 2γ group, Fig. 2d].

**Table 3:** Incidence of postoperative atrial fibrillation

Categories	Control	1γ	2γ	*p* value
All patients				
*n*	45	44	45	
POAF	11 (24.4)	8 (18.2)	5 (11.1)	0.26
Age				
70–74 years old				
*n*	25	13	21	
POAF	6 (24.0)	2 (15.4)	3 (14.3)	0.69
75–79 years old				
*n*	16	25	13	
POAF	5 (31.3)	5 (20.0)	0 (0.0)	0.09
80 years old ≦				
*n*	4	6	11	
POAF	0 (0.0)	1 (16.7)	2 (18.2)	1.00
Gender				
Male				
*n*	29	25	27	
POAF	4 (13.8)	2 (8.0)	4 (14.8)	0.76
Female				
*n*	16	19	18	
POAF	7 (43.8)	6 (31.6)	1 (5.6)	0.03
Hypertension				
No				
*n*	5	8	9	
POAF	1 (20.0)	0 (0.0)	1 (11.1)	0.69
Yes				
* n*	40	36	36	
POAF	10 (25.0)	8 (22.2)	4 (11.1)	0.28
Diabetes				
No				
*n*	36	33	39	
POAF	11 (30.6)	7 (21.2)	5 (12.8)	0.17
Yes				
*n*	9	11	6	
POAF	0 (0.0)	1 (9.1)	0 (0.0)	1.00
Dyslipidemia				
No				
*n*	27	29	22	
POAF	9 (33.3)	5 (17.2)	4 (18.2)	0.29
Yes				
*n*	18	15	23	
POAF	2 (11.1)	3 (20.0)	1 (4.3)	0.35
CVD				
No				
*n*	33	32	33	
POAF	9 (27.3)	5 (15.6)	3 (9.1)	0.14
Yes				
*n*	12	12	12	
POAF	2 (16.7)	3 (25.0)	2 (16.7)	1.00
Hemodialysis				
No				
*n*	42	40	44	
POAF	9 (21.4)	7 (17.5)	4 (9.1)	0.28
Yes				
*n*	3	4	1	
POAF	2 (66.7)	1 (25.0)	1 (100.0)	0.49
Preoperative use of β-blockers				
No				
*n*	27	20	24	
POAF	5 (18.5)	3 (15.0)	2 (8.3)	0.63
Yes				
*n*	18	24	21	
POAF	6 (33.3)	5 (20.8)	3 (14.3)	0.38
Preoperative use of ARBs				
No				
*n*	15	15	19	
POAF	8 (53.3)	3 (20.0)	2 (10.5)	0.02
Yes				
*n*	30	29	26	
POAF	3 (10.0)	5 (17.2)	3 (11.5)	0.72
Preoperative use of CCB				
No				
*n*	18	14	22	
POAF	4 (22.2)	4 (28.6)	2 (9.1)	0.27
Yes				
*n*	27	30	23	
POAF	7 (25.9)	4 (13.3)	3 (13.0)	0.44
Cardiovascular surgeries				
Valve				
*n*	16	17	18	
POAF	8 (50.0)	1 (5.9)	0 (0.0)	< 0.01
CABG				
*n*	9	6	4	
POAF	2 (22.2)	4 (66.7)	2 (50.0)	0.29
TEVAR				
*n*	15	13	13	
POAF	1 (6.7)	0 (0.0)	0 (0.0)	1.00
Aorta				
*n*	5	8	10	
POAF	0 (0.0)	3 (37.5)	3 (30.0)	0.41

All data are given as *n* (%). Subgroup analysis was performed according to age, gender, comorbidities, preoperative medications, and types of cardiovascular surgery

*γ* μg/kg/min, *POAF* postoperative atrial fibrillation, *CVD* cerebrovascular disease, *ARBs* angiotensin II receptor blockers, *CCB* calcium channel blocker, *CABG* coronary artery bypass grafting, *TEVAR* thoracic endovascular aortic repair

### Changes in echocardiographic and blood biochemical data

**Fig. 3:**
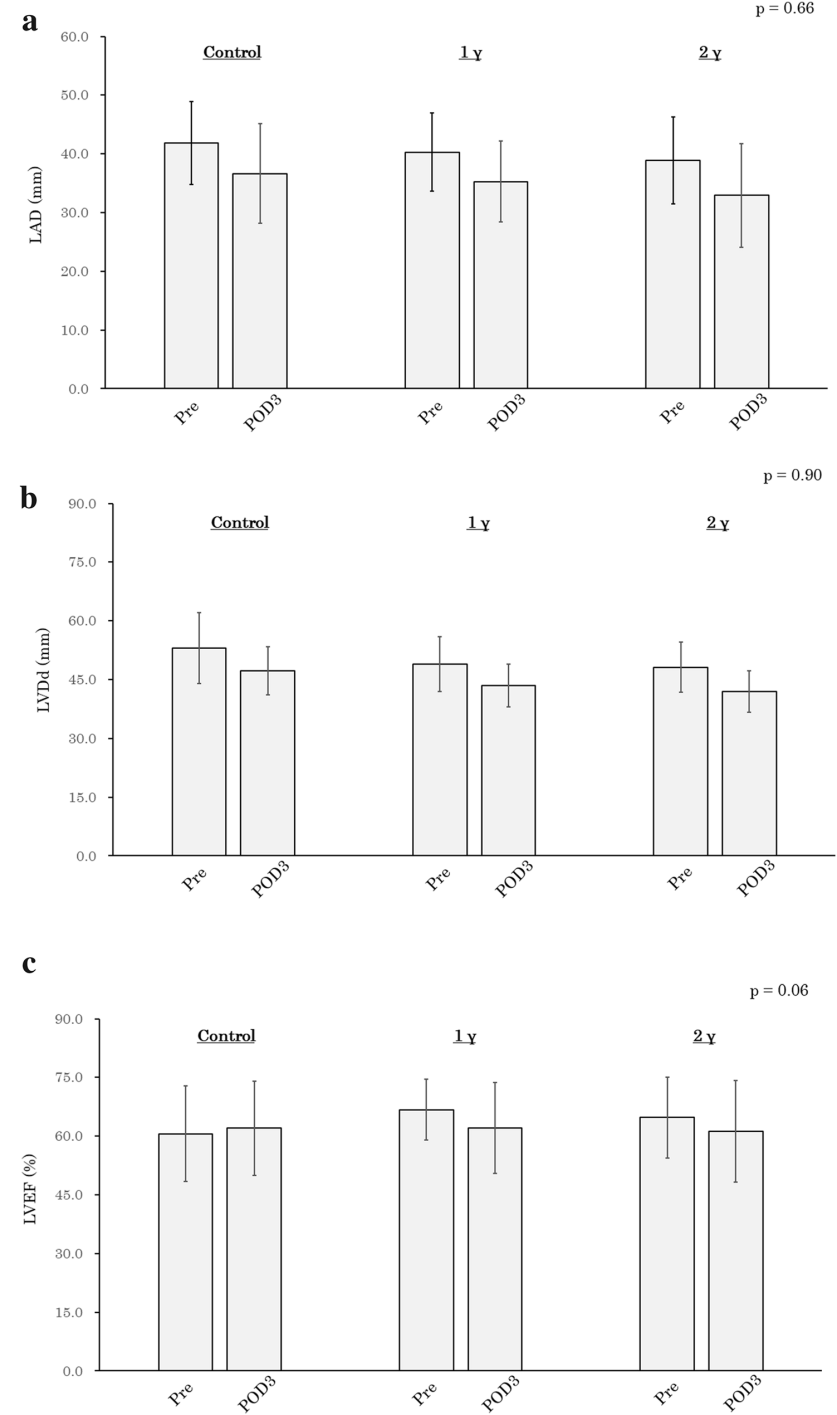

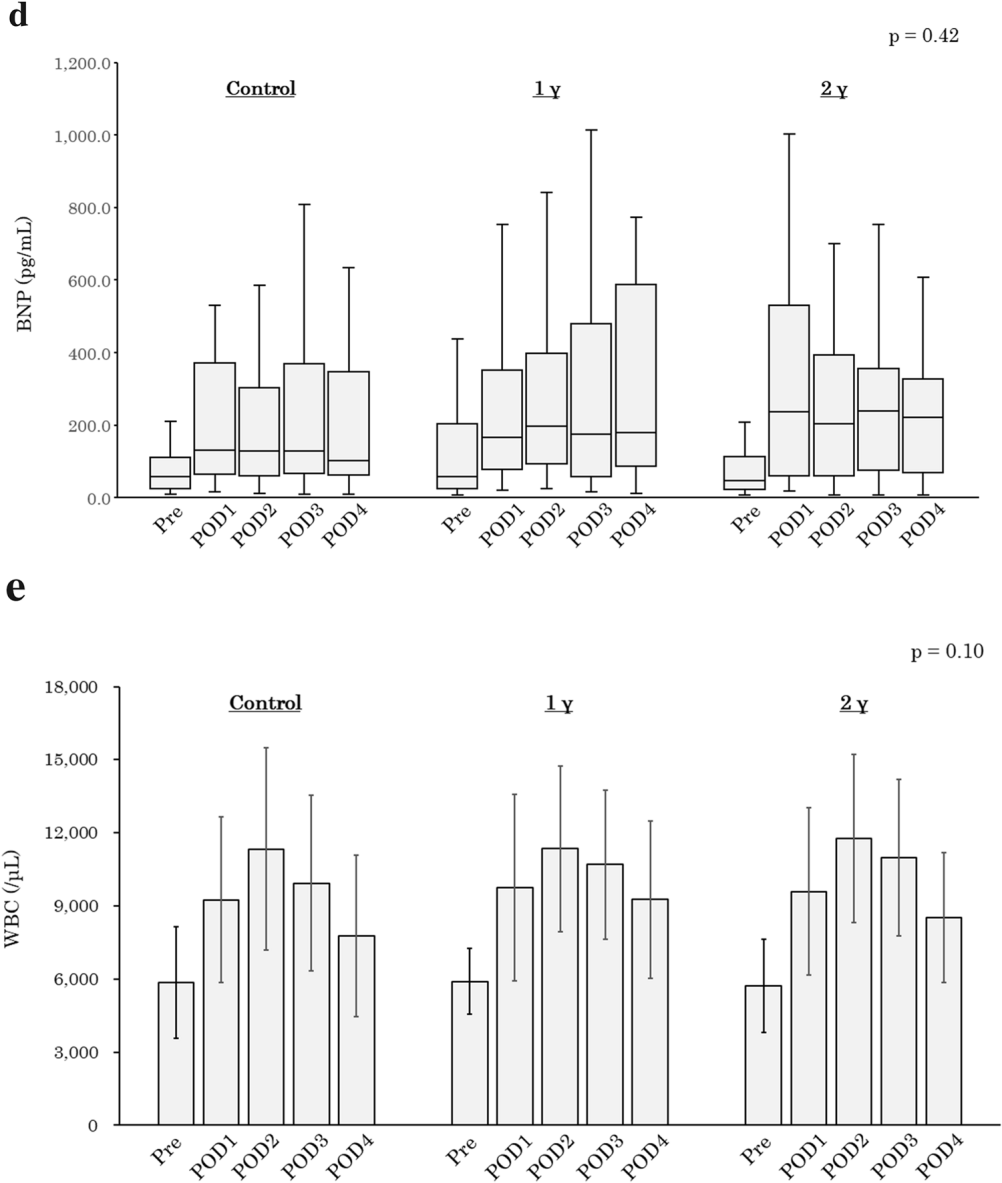
Time course of changes in echocardiographic and blood biochemical data. **a** LAD, **b** LVDd, **c** LVEF, **d** BNP, **e** WBC. All *p* values were evaluated by one-way repeated analysis of variance. Values in **a**, **b**, **c**, and **e** are mean ± standard deviation. Values of BNP are graphed as box-and-whisker plot in the appropriate format. *γ* μg/kg/min, *LAD* left atrial diameter, *LVDd* left ventricular end-diastolic diameter, *LVEF* left ventricular ejection fraction, *BNP* brain natriuretic peptide, *WBC* white blood cell, *Pre* preoperative day, *POD* postoperative day

Figure 3 shows the time course of changes in LAD, LVDd, LVEF, BNP, and white blood cell count among the three groups. The downward trends of LAD and LVDd from Pre to POD3 were not significantly different between groups (Fig. 3a and b). In addition, landiolol administration affected neither LVEF, BNP, nor white blood cell count (Fig. 3c–e).

## Discussion

Although POAF is the most commonly encountered arrhythmia after cardiovascular surgery, its underlying mechanisms of POAF have not been fully elucidated. Previous reports have demonstrated that autonomic nervous system imbalance, inflammation, and surgery-related changes in fluid volume are involved in the development of POAF [5, 12]. Postoperative neurohumoral hyperactivation, especially sympathetic overactivation, can induce calcium overload from the sarcoplasmic reticulum, resulting in delayed after-depolarization and triggered activity, which may contribute to the onset of POAF [13]. Therefore, perioperative administration of β-blocker, antagonizing the sympathetic hyperactivity, is recommended to suppress the tachyarrhythmia [14]. However, there are few reports on prophylactic β-blocker administration after CABG, valve surgery, and aortic surgery including endovascular repair. In addition, adequate standard dose of β-blockers for prophylactic use has not been established. This study demonstrated that prophylactic low-dose intravenous landiolol administration significantly decreased the incidence of POAF in female patients, those that were not on ARBs prior to surgery, and those who underwent valve surgery.

It has been reported that sympathetic nerve activity increases with aging and pronounced influence of age on sympathetic nerve activity is observed in females compared to males [15]. Age-dependent alteration in estrogens levels are one of the reasons for sympathetic hyperactivation in postmenopausal females [16]. In a recent study, fluorine-18-Dihydroxyphenylalanine positron emission tomography imaging revealed the predominance of cardiac sympathetic nerve activity in the left ventricle apical region in females > 55 years of age without cardiovascular diseases (CVD) compared with age-matched males [17]. Another study also showed that in CVD patients with or without chronic heart failure, cardiac specific sympathetic activation was higher in females than in males [18]. In our study, POAF in the control group was more frequently observed in female patients than in male patients (Table 3). This might be attributed to sympathetic overactivation in female patients. Taking into account the accumulated findings from the previous studies and our present results, we speculate that β-blockers are possibly more effective for preventing POAF in elderly females. In fact, there is additional evidence that suggests females can stand to benefit more from β-blockers. The beneficial effects of β-blockers on all-cause mortality in patients with heart failure (HF) have been clearly established in three large randomized trials: the Metoprolol CR/XL Randomized Intervention Trial in Heart Failure (MERIT-HF) [19], the Cardiac Insufficiency Bisoprolol Study (CIBIS-II) [20], and the Carvedilol Prospective Randomized Cumulative Survival Study (COPERNICUS) [21]. Of note, in MERIT-HF, the treatment with metoprolol (a β1 selective β-blocker) reduced all-cause mortality and all-cause hospitalizations in females with HF and impaired LVEF [19]. The CIBIS II also demonstrated that bisoprolol (a β1 selective β-blocker) reduced the mortality risk to a greater degree in females with HF than in males (0.52 and 0.71, respectively) [20]. Based on these studies, there is a possibility that the low-dose administration of landiolol can preferentially prevent POAF in female patients since it is also a highly cardioselective β1 blocker, although the primary endpoint of the previous study was different from ours.

Inhibitors of the renin-angiotensin system, such as angiotensin-converting enzyme inhibitors and ARBs, prevented the generation of primary atrial fibrillation in patients with left ventricular hypertrophy and/or HF [22]. Sezai et al. revealed that the pre- and postoperative maximum angiotensin II levels in CABG patients with new onset of POAF was significantly higher than in those without POAF [23]. Since angiotensin II not only induces electrophysiological and structural remodeling but is also associated with the pathogenesis of POAF, it could be speculated that the preoperative use of renin-angiotensin system blockers is beneficial for preventing POAF in patients with CVD. However, a recent meta-analysis of 11 trials incorporating 27,885 patients demonstrated that preoperative treatment with inhibitors of renin-angiotensin system did not provide additional benefits in POAF reduction after cardiac surgery [24]. In our finding, there was a landiolol dose-dependent effect on the reduction of POAF in patients with disuse of ARBs prior to surgery, whereas no positive effect was seen in patients using ARBs preoperatively. The reason for this result remains unexplored, and the interrelationship between the renin-angiotensin aldosterone system and the sympathetic nervous system has not completely elucidated, especially that in the postoperative period after cardiovascular surgery. However, it is well known that beta1-adrenergic receptors activation promotes renin release from juxtaglomerular epithelioid cells in the kidney. In addition, angiotensin II receptors type1 is located at the sympathetic nerve endings, and angiotensin II increases sympathetic nerve activity in various organs [25]. Therefore, there is a possibility that landiolol, an ultra-short-acting highly selective β 1-blocker, may inhibit resultant sympathetic overstimulation partly induced by activation of the renin-angiotensin system after surgery. Further investigations are warranted to confirm this hypothesis.

Landiolol hydrochloride has also been reported to exert an anti-inflammatory role [26]. Postoperative high-sensitivity C-reactive protein (CRP) and inflammatory biomarkers, such as interleukin (IL)-6 and IL-8, had their levels decreased by the administration of landiolol in patients undergoing CABG [27, 28]. Hagiwara et al. demonstrated that landiolol hydrochloride could attenuate the serum levels of inflammatory mediators and suppress the inflammatory response in a rat model of endotoxin-induced systemic inflammation [29]. Since the surgery-related hyperinflammatory state likely plays a role in POAF, landiolol might also be involved in reducing its risk due to its anti-inflammatory properties. However, such an anti-inflammatory effect by landiolol was not evident in our present clinical study.

POAF after valvular heart surgery is associated with intraoperative factors, primarily surgical atrial injury and atrial ischemia [5]. Compared with patients after CABG, a high incidence of POAF is observed in patients after valvular surgery, reaching up to approximately 60% [1]. In our sub-analysis according to surgical classification, POAF occurred in 8 (50.0%) patients assigned to the control group. However, few patients developed a new onset of POAF in the 1γ and 2γ landiolol groups. This POAF preventive effect is consistent with a previous study [30]. In a single-center RCT including 60 patients with valve diseases, Sakaguchi et al. documented that the incidences of the arrhythmia after surgery in the landiolol and control groups were 20.0% and 53.3%, respectively, although the average minimum dose of landiolol was nearly 7 μg/kg/min [30]. From our present study, we can infer that low-dose landiolol treatment is effective for preventing POAF in patients who undergo valvular heart surgery. Some investigators have shown that valvular heart disease is more frequently observed in elderly females [31, 32]. In our present study, more than half of the patients with valvular disease were female. Therefore, it is difficult to completely exclude the interrelationship between female sex and valvular disease; however, the prophylactic efficacy of landiolol for prevention of POAF is likely greater in cardiovascular patients exhibiting either or both characteristics, as the incidence of POAF varied in each sub-category.

Our study did not prove significant efficacy of landiolol administration for prevention of POAF onset in the CABG patients unlike in the patients undergoing valvular surgery. We speculate that one of the possible reasons could be attributed to the difference in the proportions of hemodialysis patients. This study included dialysis dependency, and the proportion of hemodialysis patients was larger in the CABG group than the valvular surgery (26.3% in CABG, 4.1% in valvular surgery). Additionally, POAF occurred in 60% of hemodialysis patients who underwent CABG, even though landiolol was administered according to the protocol corresponding to the assigned group. In isolated CABG patients with mild renal dysfunction (Creatinine 1.61 ± 0.2 mg/dl), the incidence of POAF was fivefold compared with the patients without mild renal dysfunction [33]. Thus, renal failure could likely be a confounding factor related to the effectiveness of landiolol administration.

The effect of continuous low-dose landiolol infusion on blood pressure has been documented to be small [14], whereas it has not fully examined whether or not landiolol administration affect cardiac function in an acute phase in patients undergoing cardiovascular surgery. Our findings demonstrated no significant changes in LAD, LVDd and LVEF between groups, which indicates that low-dose landiolol hydrochloride could be administered safely without exacerbation of cardiac dysfunction in an acute phase after cardiovascular surgery.

### Limitations

As with any study, there are some limitations to our clinical design. First, this study was a single-center study. Second, the number of patients enrolled was smaller than we expected. Patients undergoing TEVAR comprised 30.6% of our overall cohort and around 30% in each group, more than a quarter occupied. In addition, POAF seldom occurred in all TEVAR patients as well as each study group among TEVAR (Table 3). Hence, the prophylactic effect of the postoperative use of landiolol hydrochloride for POAF was possibly underestimated. Furthermore, the number of patients with coronary artery disease or aortic disease was relatively small compared with those who underwent valve heart surgery and TEVAR. Further multicenter studies excluding TEVAR cases are required to precisely elucidate the preventive effect of low-dose landiolol in patients with CVD in more detail. Third, the anti-inflammatory effect of landiolol hydrochloride administration was not assessed, since proinflammatory cytokines and high-sensitive CRP are not routinely measured in clinical practice.

## Conclusion

Low-dose landiolol administration may not be effective for reduction in new onset of POAF in overall patients who undergo cardiovascular surgery. However, among patients with cardiovascular disease, its prophylactic administration could have an advantage to prevent POAF onset in female patients, patients not using ARBs preoperatively, and those undergoing valvular surgery.
